# Association between the Intake of Fermented Soy Products and Hypertension Risk in Postmenopausal Women and Men Aged 50 Years or Older: The Korea National Health and Nutrition Examination Survey 2013–2018

**DOI:** 10.3390/nu12123621

**Published:** 2020-11-25

**Authors:** Dohyun Yoo, Yongsoon Park

**Affiliations:** Department of Food and Nutrition, Hanyang University, 222 Wangsimni-ro, Seongdong-gu, Seoul 04763, Korea; dptnsla102@naver.com

**Keywords:** blood pressure, elderly population, fermented soy products, hypertension, salt intake

## Abstract

Sodium intake is positively associated with hypertension risk; however, it is not clear whether there is an association between the intake of fermented soy products, a major source of salt, and blood pressure (BP). This study aimed to investigate the hypothesis that hypertension risk and BP were negatively associated with the intake of fermented soy products but not with the intake of sodium from fermented soy products. This cross-sectional study was performed using data from the Korea National Health and Nutrition Examination Survey (2013–2018). In total, 11,566 men and postmenopausal women aged ≥50 years were divided according to quintiles of sodium or fermented soy product intake. The intake of fermented soy products was negatively associated with hypertension risk (odds ratio: 0.81, 95% confidence interval: 0.66–0.98; *p*-trend = 0.023) and systolic BP (SBP; *p*-trend = 0.043) in postmenopausal women. Mediation analysis showed that the intake of fermented soy products had total and direct effects on SBP; however, there was no indirect effect because soy nutrients, such as protein, fiber, calcium, and potassium, had no significant effects on SBP. Among men, fermented soy product intake was not associated with hypertension risk and BP. Additionally, the intake of sodium from fermented soy products was not significantly associated with hypertension risk and BP in both postmenopausal women and men. This study suggests that hypertension risk and BP were not associated with the intake of sodium from fermented soy products; further, hypertension risk and BP were inversely associated with fermented soy product intake in postmenopausal women. Further clinical studies are needed to confirm the effect of fermented soy product intake on hypertension risk and BP.

## 1. Introduction

Hypertension is an important risk factor for cardiovascular diseases, which are the major cause of death globally [1]. Blood pressure (BP) increases gradually with age in men and postmenopausal women, and the prevalence of hypertension has been estimated to be approximately 65% among Korean elderly people [2].

Sodium intake is a major modifiable lifestyle risk factor for hypertension [3], and the World Health Organization recommends the consumption of <2000 mg/day of sodium to decrease BP [4]. Most epidemiological studies have reported that salt intake is positively associated with BP and the risk of hypertension [5]. Additionally, a recent meta-analysis of clinical trials showed that a reduction in sodium intake decreased BP in both participants with normotension and hypertension [6]. However, salt from miso, a traditional fermented soy product consumed in Japan, did not increase BP in salt-sensitive rats (compared to a same amount of salt) [7,8]. The use of Doenjang, a Korean fermented soy product, also resulted in lower BP in normotensive rats (compared to the use of the same amount of salt) [9].

The Korea National Health and Nutrition Examination Survey (KNHANES) VI-2 reported that the average daily sodium intake was 5700 mg/day, and the major sources of dietary sodium were table salt, kimchi, and fermented soy products such as soy sauce (Kanjang), soybean paste (Doenjang), and red pepper paste (Gochujang) [2]. Traditional Korean fermented soy products are made using sea salt [10], which not only contains sodium but also contains minerals, such as potassium and calcium [11]. Baek et al. (2015) [12] reported that the use of sea salt decreased BP; however, the use of refined salt did not decrease BP in elderly Korean patients with hypertension.

Furthermore, soybean contains nutrients such as protein, fiber, calcium, and potassium [13]. Previous studies have reported that the supplementation of soy protein [14], calcium [15], potassium [16], and fiber [17] reduced BP in participants with normotension or hypertension. In addition, Nozue et al. (2017) [18] reported that the intake of miso and natto, but not tofu, was negatively associated with the risk of hypertension, defined as systolic blood pressure (SBP) ≥130 mmHg or diastolic blood pressure (DBP) ≥85 mmHg in the Japanese population; this suggests that the intake of fermented soy products had a greater effect on BP than the intake of soy products. Kanda et al. (1999) [19] showed that the consumption of more than two bowls of miso soup per day was negatively associated with the risk of hypertension in elderly Japanese individuals. Although Ito et al. (2017) [20] reported that BP was not significantly different between the groups consuming high and low amounts of miso soup, heart rates were lower in participants of the former group; this suggests that miso intake could attenuate salt-induced BP elevation by lowering heart rate. However, no study has investigated the association between the intake of traditional Korean fermented soy products and hypertension risk and BP. Thus, this study aimed to evaluate the hypothesis that hypertension risk and BP were negatively associated with the intake of fermented soy products but were not associated with the intake of sodium from fermented soy products. In addition, this study determined whether there were direct and indirect relationships between BP and fermented soy product intake.

## 2. Materials and Methods

### 2.1. Study Population

This study was based on data from the Korea National Health and Nutrition Examination Survey (KNHANES) VI and VII (2013–2018). The KNHANES is a cross-sectional and nationally representative survey using a stratified and multistage sampling design for the selection of household units. Health interviews and health examinations were conducted by trained medical staff members at mobile examination centers, and the in-person household nutrition survey was conducted by dietitians [21].

**Figure 1:**
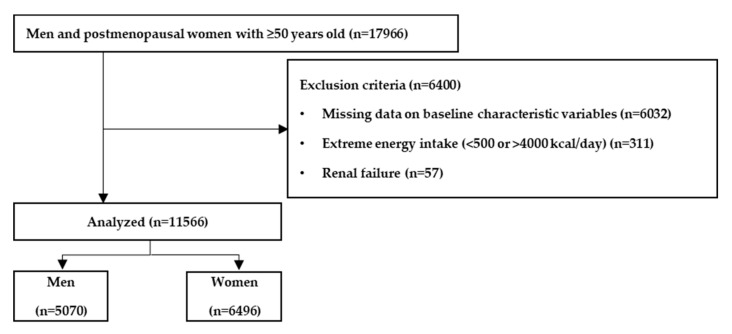
Flowchart of the inclusion and exclusion of participants.

Out of a total of 47,217 participants, there were 17,966 men and postmenopausal women aged ≥50 years (Figure 1). The exclusion criteria were as follows: missing data on baseline variables (n = 6032), extreme energy intake (<500 kcal/day or >4000 kcal/day; n = 311), or renal failure (n = 57). Extreme energy intake was defined as age and sex specific total energy intake less than 25% of estimated energy requirements (EER) or more than 200% of EER. After exclusion, 11,566 participants were included in the final analysis.

### 2.2. Data Collection

A health interview was performed to obtain information on age, sex, socioeconomic status, smoking status, drinking status, exercise status and menstruate state. Exercise was defined as walking or strength exercises for >20 min at a time at least 3 times/week or walking or moderate exercise for >30 min at a time at least 5 times/week. Drinking status was categorized into non/light-alcohol drinker or heavy alcohol drinker: men who drank ≥7 alcoholic beverages and women who drank ≥5 alcoholic beverages at a time ≥2 times a week [22]. Postmenopausal status was defined based on a self-reported questionnaire survey containing question for the determination of whether 1 year had passed since the time of last menstruation including hysterectomy.

The intakes of energy, nutrients, and fermented soy products were determined using 1-day 24-h recall and were calculated by multiplying the food code-specific nutrient concentration data by the corresponding weight of each reported food. All the reported items were coded using the Korea Food Composition Table for the KNHANES [23,24], which provided data on nutritional content based on standardized recipes. Fermented soy products include Doenjang (soybean paste fermented for 3–6 month), Chongkukjang (soybean paste fermented for 2–3 days), Kanjang (fermented soy sauce), and Gochujang (fermented red pepper paste) [10].

A health examination survey was performed to obtain information on the levels of fasting plasma glucose (FPG) and lipid profiles (triglyceride, low-density lipoprotein, and high-density lipoprotein) measured directly using a Hitachi Automatic Analyzer 7600 (Hitachi, Tokyo, Japan). The Friedewald equation was used to calculate low-density lipoprotein cholesterol (LDL-C) levels for participants with no data on LDL-C levels [25]. Body mass index (BMI) was calculated using the measured height and weight. BP was measured thrice by trained technicians in the right arm using a standard mercury sphygmomanometer (Baumanometer; W. A. Baum, Copiague, NY, USA). The average of the second and third readings was considered as the final BP. Hypertension was defined as SBP ≥140 mmHg, DBP ≥90 mmHg, or the current use of antihypertensive medications [26].

### 2.3. Statistical Analysis

All statistical analyses of complex sample survey data were performed using Statistical Package for the Social Sciences (SPSS) version 25.0 (SPSS Inc., Chicago, IL, USA). To ensure that the dataset represented the entire Korean population without biased estimates, sampling weights were applied to each participant’s data [27]. The baseline characteristics and risk factors for hypertension in the study population were compared using Student’s t-test for continuous variables and the chi-square test for categorical variables. Continuous variables were presented as means and standard errors of the mean, and categorical variables were presented as frequencies and percentages. All groups were subdivided into five groups according to quintiles of sodium and fermented soy product intakes. Analysis of covariance (ANCOVA) with a Bonferroni’s post hoc test was used to assess mean differences in BP among the intake quintiles following adjustment for confounders. Multivariate logistic regression models were performed to examine the associations between the intakes of sodium and fermented soy products and the risk for hypertension. The covariates showing *p*-values <0.20 in the multivariate models were selected as the confounders and included in the fully adjusted model [28]. For men, the covariates were age, LDL-C levels, energy intake, FPG levels, BMI, education levels, drinking status, exercise status, and family history of hypertension; for women, the covariates were age, triglyceride (TG) levels, LDL-C levels, energy intake, FPG levels, BMI, education levels, drinking status, household income, and family history of hypertension.

The *p*-value for trend was calculated using multivariate logistic regression analyses by handling the median value for each category of sodium intake and fermented soy product intake as a continuous value. A mediation analysis was conducted using the Hayes PROCESS macro (Model 4) to test whether the relationship between fermented soy product intake and BP was mediated by various nutrients [29]. The significance of the mediated effect was evaluated by calculating bias-corrected bootstrap 95% confidence intervals (95% CIs). If the 95% CI did not include zero, the criteria for mediation were met. All statistical tests were two-sided according to a significance level of <0.05.

## 3. Results

### 3.1. Characteristics of the Participants

It was observed that men and postmenopausal women with normotension were younger, had lower BMI, lower blood levels of TG, FPG, SBP, and DBP, and a lower prevalence of family history of hypertension and alcohol drinking than those with hypertension but had higher education levels, income, LDL-C levels, and high-density lipoprotein cholesterol (HDL-C) levels (Table 1).

The number of smokers was higher in men with normotension than in men with hypertension, and the number of participants who exercised regularly was higher in women with normotension than in women with hypertension. The energy was higher in men and women with normotension than in those with hypertension, but intake of sodium from fermented soy product was not different between normotension and hypertension in both men and women. Intake of total sodium and fermented soy products was higher in women with normotension than in women with hypertension. However, intake of total sodium and sodium from fermented soy product was not different between normotension and hypertension in men.

### 3.2. Associations Between the Risk of Hypertension and Intakes of Sodium and Fermented Soy Products

The intakes of total sodium and sodium from fermented soy products were negatively associated with the risk of hypertension in women before, but not after, adjusting for confounders. However, the intake of fermented soy products was negatively associated with the risk of hypertension in women before and after adjusting for confounders (Table 2). In addition, the intakes of total sodium, sodium from fermented soy products, and fermented soy products were negatively associated with the risk of hypertension in men; however, the association disappeared after adjusting for confounders (Table 3).

### 3.3. Associations Between Blood Pressure and the Intakes of Sodium and Fermented Soy Products

**Figure 2:**
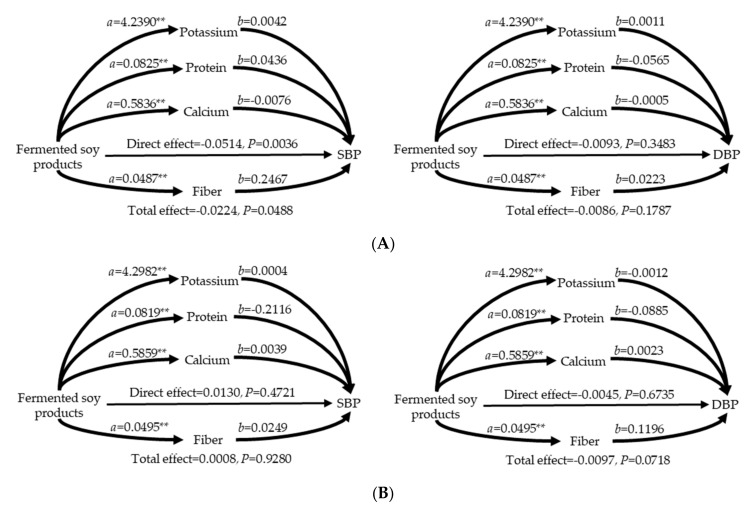
Mediation effects of nutrients on the association between the intake of fermented soy products and blood pressure in postmenopausal women (**A**) and men (**B**) DBP, diastolic blood pressure; SBP, systolic blood pressure. The confounders were age, triglyceride levels, low-density lipoprotein cholesterol levels, energy intake, fasting plasma glucose levels, body mass index, education levels, drinking status, household income, and family history of hypertension in women and age, low-density lipoprotein cholesterol levels, energy intake, fasting plasma glucose levels, body mass index, education levels, drinking status, exercise status, and family history of hypertension in men. Unstandardized coefficients are shown along with their estimated *p* values using the bootstrapping method. ** *p* < 0.001.

Among women, SBP was negatively associated with the intake of fermented soy products after adjustment for confounders; however, DBP did not show this association (Table 4). In addition, the intakes of total sodium and sodium from fermented soy products were not associated with either SBP or DBP after adjusting for confounders. In men, the intakes of total sodium, sodium from fermented soy products, and fermented soy products were not associated with both SBP and DBP after adjusting for confounders (Table 5). Among postmenopausal women, mediation analysis showed that the intake of fermented soy products had a total and direct effect on SBP but not on DBP; additionally, any nutrients from fermented soy products had no indirect effect (Figure 2, Table 6). Moreover, there were no total, direct, or indirect effects of the intake of fermented soy products on SBP and DBP among men.

## 4. Discussion

This study shows that the intake of fermented soy products was negatively associated with the risk of hypertension and SBP in postmenopausal women aged ≥50 years but not in men. Consistent with the present study, Kanda et al. (1999) [19] reported that the intake of miso soup (>2 bowls per day) was negatively associated with the risk of hypertension during a 4-year follow-up study in elderly Japanese individuals. Nouze et al. (2017) [18] also reported that the intake of miso or natto was negatively associated with the risk of elevated BP (SBP ≥130 mmHg or DBP ≥85 mmHg) during a 5-year follow-up study in middle-aged Japanese individuals. In addition, dietary patterns characterized by high intakes of natto and tofu were negatively associated with the risk of elevated BP in Japanese individuals [30]. Furthermore, soy intake has been reported to be negatively associated with the risk of hypertension in the China Health and Nutrition Survey [31] and the Korean Multi-Rural Communities Cohort study [32].

The intake of fermented soy products was also negatively associated with SBP in the present study. Ito et al. (2017) [20] reported that there were no significant differences in BP among subjects in the four quartiles of consumption frequency of miso soup (highest quartile: ≥1 bowl per day). Kanda et al. (1999) [19] reported that the consumption of two bowls of miso soup per day was negatively associated with the risk of hypertension; however, the consumption of ≤1 bowl of miso soup per day was not, suggesting that the consumption of at least two bowls of miso soup could have an effect on BP. The present study shows that the intake of fermented soy products in the highest quintile was >26 g, which corresponds to >24 g of miso (present in two bowls of miso soup). Furthermore, previous epidemiologic studies have consistently reported that the intake of soy food was inversely associated with BP in Chinese [33] and American individuals [34,35]. In addition, the supplementation of soy products, such as soy milk [36] and soy nuts [37,38], reduced BP in participants with normotension and hypertension, suggesting that soy nutrients might help decrease BP. Soy contains protein, fiber, and calcium, and potassium [13]. Dietary Approaches to Stop Hypertension diet is an appropriate diet for hypertension, since the diet is rich in protein, fiber, calcium, and potassium [39]. Clinical trials also have shown that BP was reduced in response to supplementation with soy protein [14], fiber [17], calcium [15], and potassium [16] in participants with normotension and hypertension. In the present study, the intakes of protein, fiber, calcium, and potassium from fermented soy products were 1.65 g/day, 0.88 g/day, 11.13 mg/day, and 94 mg/day, respectively. However, those nutrients were only 0.03%, 0.04%, 0.01%, and 0.03% of Korea Dietary Recommended Intake.

In this study, we performed mediation analysis to confirm the indirect effects of soy nutrients on BP; the intake of fermented soy products showed no indirect effects via soy nutrients. During the fermentation of soy, the level of peptides increases [40], and the glycoside form of isoflavone is transformed to aglycone forms, such as daidzein and genistein [41]. Aglycones of isoflavone had a beneficial effect on hepatic lipid metabolism compared with both glycosides of isoflavone and an isoflavone-free diet, indicating that aglycones of isoflavone showed bioavailability [42]. Shin et al. (2001) [43] reported that the injection of isolated peptides from Doenjang inhibited the activity of angiotensin-converting enzyme and thus reduced SBP in spontaneously hypertensive rats. In addition, daidzein and genistein enhanced the activity of endothelial nitric oxide synthase, which improved vascular function and thus reduced SBP in spontaneously hypertensive rats [44]. These studies suggested that the peptide and aglycone forms of isoflavone reduced BP; however, the present study did not include the peptide and aglycone forms of isoflavone in the mediation analysis due to lack of data.

Previous epidemiologic studies have shown that sodium intake is positively associated with the risk of hypertension [45] and with BP [46]. A meta-analysis of clinical trials also showed that BP was decreased after a reduction in sodium intake in both participants with normotension and hypertension [6]. However, the present study shows that the intakes of total sodium and sodium from fermented soy products were not associated with the risk of hypertension and with BP. Fermented soy products are a major source of sodium, and their intake is positively associated with the daily intake of salt among Koreans [2,47]. Previous studies have reported that fermented soy products contain soy nutrients and sea salt, which have beneficial effects on BP [11,12]. Mun et al. (2019) [9] showed that SBP was increased in rats fed 8% salt than in those fed 0.3% salt; however, SBP was observed to be the same in rats fed Doenjang containing 8% salt and 0.3% salt, suggesting that fermented soy products could attenuate salt-induced hypertension by offsetting the side effects of salty fermented soy products.

In this study, the beneficial effects of the intake of fermented soy products on the risk of hypertension and in terms of BP were significant only in women. Yang et al. (2005) [33] showed that soy intake was negatively associated with SBP in postmenopausal women but not in premenopausal women. It is well known that estrogen lowers BP by promoting vasodilation, decreasing vascular inflammation, and improving vascular reactivity [48]; isoflavone acts as an estrogen agonist in a low-estrogen environment [49]. Hooper et al. (2009) [50] showed that supplementation with soy isoflavone increased the blood level of estradiol in postmenopausal women. However, soy intake was negatively correlated with blood estradiol levels in men [51]. Taku et al. (2010) [52] also showed that the supplementation of isoflavones (aglycone equivalents) decreased SBP in postmenopausal women, but not men, with normotension and pre-hypertension.

This study shows that soy intake had beneficial effects on SBP but on DBP. After the age of 50 years, SBP continues to rise but DBP tends to fall, and isolated systolic hypertension is predominant in elderly patients [53]. Additionally, menopause is associated with an accelerated age-related increase in the vascular stiffness of large arteries, which contributes to the rise in SBP [54]. Nestel et al. (2007) [55] also showed that isoflavone supplementation reduced pulse wave velocity, which signified arterial stiffness, and thus reduced SBP in overweight participants, suggesting that isoflavone supplementation had a greater effect on SBP than on DBP.

The major strength of this study lies in the fact that data were gathered from a nationally representative survey; thus, the findings could be generalized to the Korean population. However, the present study has a few limitations. First, due to the cross-sectional design of the study, we were unable to establish a cause-and-effect relationship between the intake of fermented soy products and the risk of hypertension. Second, although adjustments were made for various confounders, certain residual confounders might still remain. Third, although the validated 1-day 24-h recall method was used, 1-day 24-h recall was restricted to reflect the usual intake than the food frequency questionnaire. Finally, sodium intake was estimated by dietary recall but not by 24-h urinary sodium excretion.

## 5. Conclusions

This study demonstrates that the intake of fermented soy products with a high salt content was inversely associated with the risk of hypertension and with BP in postmenopausal women. However, sodium from fermented soy products was not associated the risk of hypertension and with BP in both men and women, suggesting that salt intake from fermented soy products might not increase hypertension risk and BP. Further clinical studies are needed to confirm the effect of the intake of fermented soy products on the risk of hypertension and on BP.

## Figures and Tables

**Table 1:** Baseline characteristics of men and postmenopausal women aged ≥50 years.

Variables	Women		*p*–Value	Men		*p*–Value
	Normotensive (*n* = 3438)	Hypertensive (*n* = 3058)		Normotensive (*n* = 2655)	Hypertensive (*n* = 2415)	
Age (years)	60.07 ± 0.16	65.93 ± 0.19	<0.001	59.72 ± 0.18	63.26 ± 0.23	<0.001
BMI (kg/m^2^)			<0.001			<0.001
<18.5	97 (2.9)	34 (1.2)		71 (2.4)	43 (1.6)	
18.5 to <23.0	1504 (45.2)	808 (27.6)		1039 (37.6)	663 (26.1)	
23.0 to <25.0	871 (25.0)	793 (24.7)		775 (29.7)	630 (26.5)	
≥25.0	966 (27.0)	1423 (46.4)		770 (30.3)	1079 (45.8)	
Family history of hypertension, *n* (%)	964 (29.6)	1166 (40.9)	<0.001	588 (25.8)	815 (37.2)	<0.001
Regular exercise, *n* (%) ^1^	1594 (48.1)	1140 (39.5)	<0.001	1262 (48.4)	1078 (46.0)	0.149
Alcohol drinking, *n* (%) ^2^	50 (1.5)	68 (2.6)	0.007	262 (11.2)	364 (18.0)	<0.001
Smoking, *n* (%)			0.781			0.020
Never	3263 (94.8)	2897 (94.7)		521 (19.1)	455 (19.2)	
Past	80 (2.3)	81 (2.6)		1343 (48.4)	1343 (52.5)	
Current	95 (3.0)	80 (2.8)		791 (32.5)	617 (28.4)	
Education level, *n* (%)			<0.001			<0.001
≤ Elementary school	1191 (30.2)	1823 (55.2)		617 (18.7)	686 (25.2)	
Middle school	658 (19.4)	467 (16.3)		425 (14.6)	447 (17.0)	
High school	1040 (33.3)	540 (20.1)		820 (32.7)	752 (32.4)	
≥College	549 (17.2)	228 (8.4)		793 (34.0)	530 (25.4)	
Household income, *n* (%)			<0.001			<0.001
Low	753 (19.1)	1200 (36.1)		514 (15.0)	663 (22.9)	
Middle-low	917 (25.2)	808 (25.2)		697 (23.6)	644 (24.9)	
Middle-high	806 (25.0)	590 (21.3)		654 (27.1)	542 (24.3)	
High	962 (30.7)	460 (17.5)		790 (34.3)	566 (27.9)	
LDL-C (mmol/L)	3.29 ± 0.02	2.98 ± 0.02	<0.001	3.06 ± 0.02	2.73 ± 0.02	<0.001
HDL-C (mmol/L)	1.39 ± 0.01	1.31 ± 0.01	<0.001	1.21 ± 0.01	1.19 ± 0.01	0.057
TG (mmol/L)	1.38 ± 0.02	1.56 ± 0.02	<0.001	1.75 ± 0.04	1.88 ± 0.04	0.011
FPG (mmol/L)	5.48 ± 0.02	5.95 ± 0.03	<0.001	5.81 ± 0.03	6.13 ± 0.04	<0.001
SBP (mmHg)	114.84 ± 0.24	134.88 ± 0.39	<0.001	116.21 ± 0.26	132.42 ± 0.40	<0.001
DBP (mmHg)	72.78 ± 0.15	78.11 ± 0.26	<0.001	75.38 ± 0.18	80.52 ± 0.31	<0.001
Energy intake (kcal/day)	1681.51 ± 12.61	1569.34 ± 12.33	<0.001	2191.55 ± 16.82	2105.22 ± 17.56	<0.001
Total sodium intake (mg/day)^3^	2928.53 ± 33.92	2820.17 ± 36.30	0.017	4030.34 ± 44.21	4014.40 ± 55.26	0.819
Sodium intake from fermented soy products (mg/day) ^3^	690.48 ± 15.33	691.22 ± 15.45	0.971	924.89 ± 20.66	893.00 ± 21.27	0.260
Fermented soy product intake (g/day)	17.47 ± 0.42	16.02 ± 0.38	0.008	23.60 ± 0.59	22.32 ± 0.60	0.117

BMI, body mass index; DBP, diastolic blood pressure; FPG, fasting plasma glucose; HDL-C, high-density lipoprotein cholesterol; LDL-C, low-density lipoprotein cholesterol; SBP, systolic blood pressure; TG, triglyceride. Continuous variables are presented as means ± standard error of the mean, while categorical variables are presented as the subject number (percentage distribution); ^1^ Regular exercise was defined as vigorous activity for >20 min, >3 times a week or walking or moderate exercise for 30 min, >5 days a week; ^2^ Alcohol drinking was defined as drinking at least 7 beverages for men and at least 5 beverages for women ≥2 times a week; **^3^** Adjusted for energy intake.

**Table 2:** Associations between the risk of hypertension and the intakes of sodium and fermented soy products in postmenopausal women aged ≥50 years.

Variables	Quintiles of Intake					*p*-Trend ^1^
	Q1 (*n* = 1299)	Q2 (*n* = 1299)	Q3 (*n* = 1300)	Q4 (*n* = 1299)	Q5 (*n* = 1299)	
Total sodium intake (mg/day)	<1437.25	1437.25 to <2085.23	2085.23 to <2846.36	2846.36 to <3936.08	≥3936.08	
Crude OR (95% CI)	1	0.80 (0.67–0.96)	0.71 (0.59–0.84)	0.63 (0.53–0.76)	0.62 (0.52–0.74)	<0.001
Adjusted OR (95% CI) ^2^	1	0.88 (0.71–1.08)	0.83 (0.67–1.02)	0.82 (0.66–1.02)	0.82 (0.64–1.05)	0.183
Sodium intake from fermented soy products (mg/day)	<130.57	130.57 to <354.53	354.53 to <627.84	627.84 to <1076.44	≥1076.44	
Crude OR (95% CI)	1	0.85 (0.72–1.02)	0.82 (0.68–0.98)	0.90 (0.76–1.07)	0.76 (0.64–0.90)	0.011
Adjusted OR (95% CI) ^2^	1	0.93 (0.76–1.14)	0.92 (0.76–1.13)	1.02 (0.83–1.25)	0.83 (0.68–1.01)	0.124
Fermented soy product intake (g/day)	<2.85	2.85 to <7.89	7.89 to <14.60	14.60 to <26.39	≥26.39	
Crude OR (95% CI)	1	0.91 (0.76–1.08)	0.85 (0.71–1.02)	0.88 (0.74–1.05)	0.72 (0.61–0.86)	<0.001
Adjusted OR (95% CI) ^2^	1	0.97 (0.80–1.19)	1.00 (0.82–1.23)	1.00 (0.82–1.23)	0.81 (0.66–0.98)	0.023

CI, confidence interval; OR, odds ratio, Data are presented as ORs and 95% CIs. The ^1^ estimated *p-*trend for a linear trend was based on the linear scores derived from the median values from the quintiles of sodium intake and fermented soy product intake among women. ^2^ Adjusted for age, triglyceride levels, low-density lipoprotein cholesterol levels, energy intake, fasting plasma glucose levels, body mass index, education levels, drinking status, household income, and family history of hypertension.

**Table 3:** Associations between the risk of hypertension and the intakes of sodium and fermented soy products in men aged ≥50 years.

Variables	Quintiles of Intake					*p*-Trend ^1^
	Q1 (*n* = 1014)	Q2 (*n* = 1014)	Q3 (*n* = 1014)	Q4 (*n* = 1014)	Q5 (*n* = 1014)	
Total sodium intake (mg/day)	<2165.32	2165.32 to <3068.22	3068.22 to <4013.82	4013.82 to <5365.74	≥5365.74	
Crude OR (95% CI)	1	0.74 (0.61–0.91)	0.67 (0.55–0.83)	0.78 (0.64–0.94)	0.67 (0.54–0.82)	<0.001
Adjusted OR (95% CI) ^2^	1	0.80 (0.64–1.02)	0.78 (0.62–0.98)	0.94 (0.74–1.19)	0.77 (0.60–1.00)	0.209
Sodium intake from fermented soy products (mg/day)	<206.93	206.93 to <493.55	493.55 to <859.18	859.18 to <1469.45	≥1469.45	
Crude OR (95% CI)	1	0.96 (0.79–1.18)	0.84 (0.69–1.03)	0.88 (0.72–1.09)	0.79 (0.65–0.97)	0.020
Adjusted OR (95% CI) ^2^	1	1.03 (0.83–1.28)	0.90 (0.73–1.11)	1.01 (0.80–1.27)	0.85 (0.68–1.07)	0.150
Fermented soy product intake (g/day)	<4.66	4.66 to <11.57	11.57 to <20.62	20.62 to <36.34	≥36.34	
Crude OR (95% CI)	1	0.88 (0.72–1.07)	0.88 (0.72–1.08)	0.74 (0.60–0.92)	0.81 (0.66–1.00)	0.043
Adjusted OR (95% CI) ^2^	1	0.94 (0.76–1.17)	0.94 (0.75–1.17)	0.84 (0.66–1.05)	0.89 (0.70–1.12)	0.281

CI, confidence interval; OR, odds ratio, data are presented as ORs and 95% CIs. The ^1^ estimated *p-*trend for a linear trend was based on the linear scores derived from the median values from the quintiles of sodium intake and fermented soy product intake among men; ^2^ adjusted for age, low-density lipoprotein cholesterol levels, energy intake, fasting plasma glucose levels, body mass index, education levels, drinking status, exercise status, and family history of hypertension.

**Table 4:** Association between blood pressure and the intakes of sodium and fermented soy products in postmenopausal women aged ≥50 years.

Variables	Quintiles of Intake					*p*-Trend ^1^
	Q1 (*n* = 1299)	Q2 (*n* = 1299)	Q3 (*n* = 1300)	Q4 (*n* = 1299)	Q5 (*n* = 1299)	
Total sodium intake (mg/day)	<1437.25	1437.25 to <2085.23	2085.23 to <2846.36	2846.36 to <3936.08	≥3936.08	
SBP (mmHg)	125.44 ± 0.61	124.47 ± 0.57	123.23 ± 0.59	122.74 ± 0.58	122.20 ± 0.57	0.612
DBP (mmHg)	75.16 ± 0.33	74.79 ± 0.31	75.15 ± 0.33	74.96 ± 0.31	75.50 ± 0.31	0.838
Sodium intake from fermented soy products (mg/day)	<130.57	130.57 to <354.53	354.53 to <627.84	627.84 to <1076.44	≥1076.44	
SBP (mmHg)	124.51 ± 0.61	123.48 ± 0.55	123.91 ± 0.64	123.93 ± 0.56	122.25 ± 0.57	0.087
DBP (mmHg)	75.40 ± 0.32	75.16 ± 0.31	75.03 ± 0.33	75.24 ± 0.31	74.74 ± 0.29	0.148
Fermented soy product intake (g/day)	<2.85	2.85 to <7.89	7.89 to <14.60	14.60 to <26.39	≥26.39	
SBP (mmHg)	124.51 ± 0.60	124.05 ± 0.56	123.56 ± 0.61	123.88 ± 0.55	122.05 ± 0.58	0.043
DBP (mmHg)	75.36 ± 0.33	75.21 ± 0.31	75.05 ± 0.32	75.29 ± 0.31	74.65 ± 0.30	0.067

DBP, diastolic blood pressure; SBP, systolic blood pressure, All values are presented as means ± standard error of the mean; ^1^ *p*-trend for differences in SBP and DBP according to quintiles of sodium intake and fermented soy product intake after adjustment for confounders, such as age, triglyceride levels, low-density lipoprotein cholesterol levels, energy intake, fasting plasma glucose levels, body mass index, education levels, drinking status, household income, and family history of hypertension.

**Table 5:** Association between blood pressure and the intakes of sodium and fermented soy products in men aged ≥50 years.

Variables	Quintiles of Intake					*p*-Trend ^1^
	Q1 (*n* = 1014)	Q2 (*n* = 1014)	Q3 (*n* = 1014)	Q4 (*n* = 1014)	Q5 (*n* = 1014)	
Total sodium intake (mg/day)	<2165.32	2165.32 to <3068.22	3068.22 to <4013.82	4013.82 to <5365.74	≥5365.74	
SBP (mmHg)	125.51 ± 0.57	123.50 ± 0.60	122.23 ± 0.54	123.41 ± 0.58	122.83 ± 0.58	0.180
DBP (mmHg)	77.70 ± 0.41	77.49 ± 0.39	77.01 ± 0.36	78.59 ± 0.37	78.43 ± 0.39	0.195
Sodium intake from fermented soy products (mg/day)	<206.93	206.93 to <493.55	493.55 to <859.18	859.18 to <1469.45	≥1469.45	
SBP (mmHg)	123.90 ± 0.56	123.93 ± 0.58	122.66 ± 0.60	122.85 ± 0.57	123.96 ± 0.63	0.614
DBP (mmHg)	77.63 ± 0.40	78.16 ± 0.36	77.24 ± 0.39	77.26 ± 0.38	78.10 ± 0.38	0.304
Fermented soy product intake (g/day)	<4.66	4.66 to <11.57	11.57 to <20.62	20.62 to <36.34	≥36.34	
SBP (mmHg)	124.07 ± 0.57	123.43 ± 0.58	123.34 ± 0.57	122.24 ± 0.54	124.22 ± 0.61	0.450
DBP (mmHg)	77.74 ± 0.39	77.70 ± 0.37	77.45 ± 0.36	77.43 ± 0.39	78.07 ± 0.38	0.239

DBP, diastolic blood pressure; SBP, systolic blood pressure, All values are presented as means ± standard error of the mean; ^1^ *p*-trend for differences in SBP and DBP according to quintiles of sodium intake and fermented soy product intake after adjustment for confounders, such as age, low-density lipoprotein cholesterol levels, energy intake, fasting plasma glucose levels, body mass index, education levels, drinking status, exercise status, and family history of hypertension.

**Table 6:** Mediating effects of nutrients on the association between the intake of fermented soy products and blood pressure ^1^

	SBP		DBP	
	Women	Men	Women	Men
Indirect effect				
Protein	0.0036 (−0.0158, 0.0214)	−0.0173 (−0.0520, 0.0121)	−0.0047 (−0.0238, 0.0017)	−0.0073 (−0.0224, 0.0074)
Fiber	0.0120 (−0.0090, 0.0349)	0.0012 (−0.0216, 0.0236)	0.0011 (−0.0104, 0.0140)	0.0059 (−0.0063, 0.0190)
Calcium	−0.0045 (−0.0209, 0.0123)	0.0023 (−0.0053, 0.0348)	−0.0003 (−0.0080, 0.0093)	0.0014 (−0.0080, 0.0112)
Potassium	0.0179 (−0.0006, 0.0345)	0.0017 (−0.0182, 0.0196)	0.0046 (−0.0056, 0.0143)	−0.0052 (−0.0173, 0.0069)

DBP, diastolic blood pressure; SBP, systolic blood pressure, ^1^ Data are presented as estimated effect values and bias-corrected bootstrap 95% confidence intervals obtained after mediation analysis, using the Hayes PROCESS macro, adjusted for age, triglyceride levels, low-density lipoprotein cholesterol levels, energy intake, fasting plasma glucose levels, body mass index, education levels, drinking status, household income, and family history of hypertension in women; and adjusted for age, low-density lipoprotein cholesterol levels, energy intake, fasting plasma glucose levels, body mass index, education levels, drinking status, exercise status, and family history of hypertension in men.
